# External Ventricular Drain Migration Causing Parinaud’s Syndrome: A Case Report

**DOI:** 10.7759/cureus.10981

**Published:** 2020-10-16

**Authors:** Christine Mau, Ira Goldstein

**Affiliations:** 1 Neurosurgery, Penn State Hershey Medical Center, Hershey, USA; 2 Neurosurgery, Rutgers New Jersey Medical School, Newark, USA

**Keywords:** dorsal midbrain syndrome, external ventricular drain, neurosurgical procedures, parinaud's syndrome, vertical gaze palsy

## Abstract

Background: External ventricular drains (EVD) are used for emergent management of acute hydrocephalus and for monitoring of intracranial pressure. Common complications of EVDs include malposition, infection, and hemorrhage. Here, the authors present a novel case of EVD migration causing Parinaud’s syndrome.

Case description: A thirty-three-year-old female presented with witnessed seizure secondary to a left supraclinoid internal carotid artery aneurysm and trace subarachnoid hemorrhage. Two days after radiographic confirmation of an accurately placed EVD, she was found to have vertical gaze palsy (Parinaud’s syndrome). Repeat CT head demonstrated inward migration of the EVD with left midbrain compression. After readjustment of the EVD, her Parinaud’s syndrome improved each day until discharge home.

Conclusions: This is a novel clinical presentation of an EVD causing Parinaud’s syndrome. There is only one other case report in the literature of this phenomenon. Although a practical solution to prevent this incident from occurring is unclear, vigilance for changes in neurological exam allowed for quick assessment and revision of the EVD and subsequent recovery in this patient.

## Introduction

External ventricular drains (EVD) are used for emergent management of acute hydrocephalus (following intraventricular bleed or meningitis) and for monitoring of intracranial pressure (while managing malignant traumatic intracranial hypertension). Placement is most often performed at the bedside using the free-hand method and using surface landmarks to determine the appropriate entry point. Most commonly, Kocher’s point is used, which is at the mid pupillary line (3 centimeters lateral to midline) and approximately 11 centimeters posterior to the nasion. Head computed tomographic (CT) scan is used to confirm correct placement but more recently, there has been debate on the merit of image-guided EVD placement [1-3]. Here, we present a case of an accurately placed EVD that subsequently migrated to abut the dorsal midbrain, causing Parinaud’s syndrome.

## Case presentation

**Figure 1:**
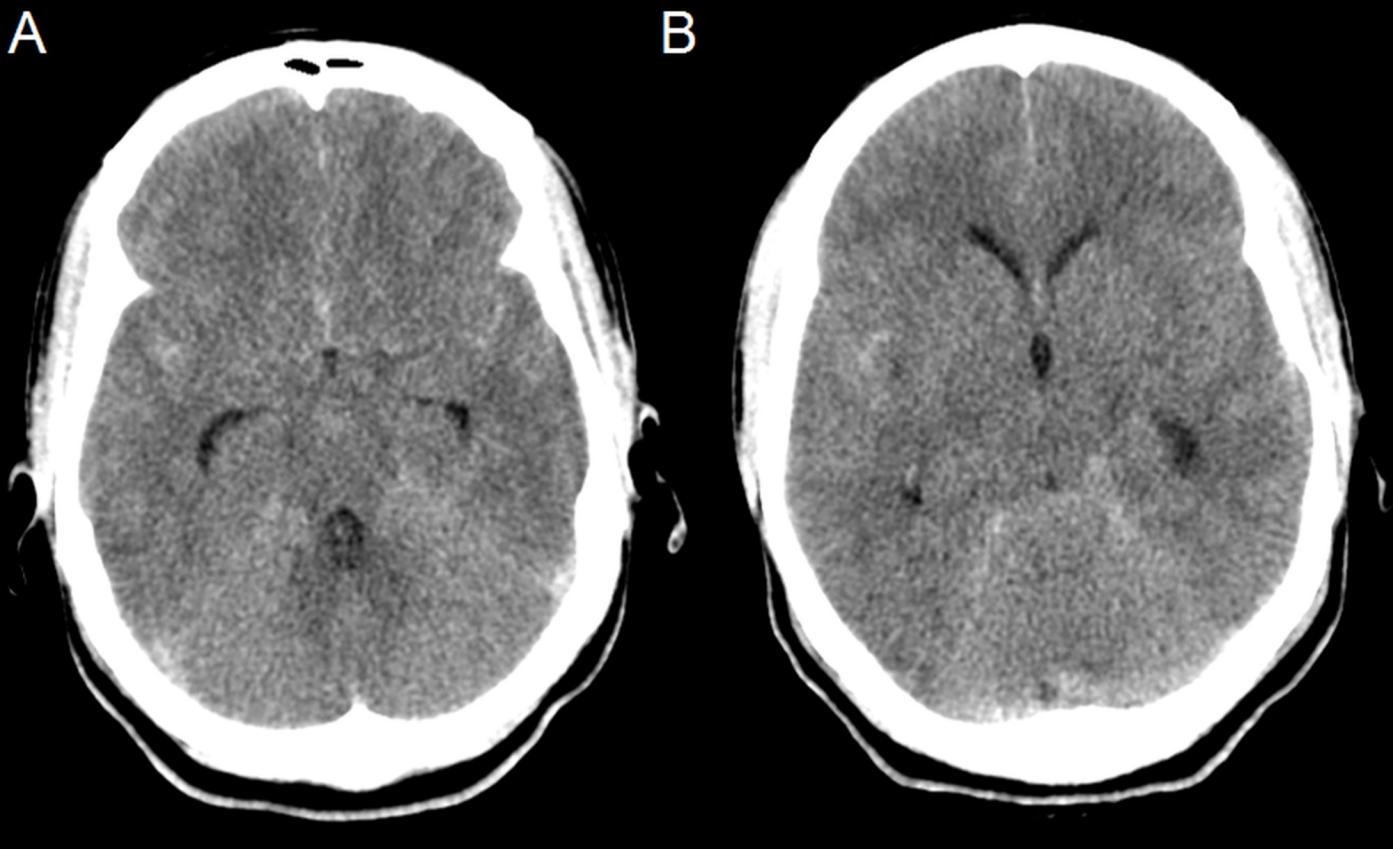
Axial pre-operative CT head scans (A and B) from outside institution showing subarachnoid hemorrhage and hydrocephalus

The patient is a thirty-three-year-old who sustained a seizure secondary to subarachnoid hemorrhage (SAH). She was taken to an outside institution where she complained of headache and nausea (Figure 1). 

**Figure 2:**
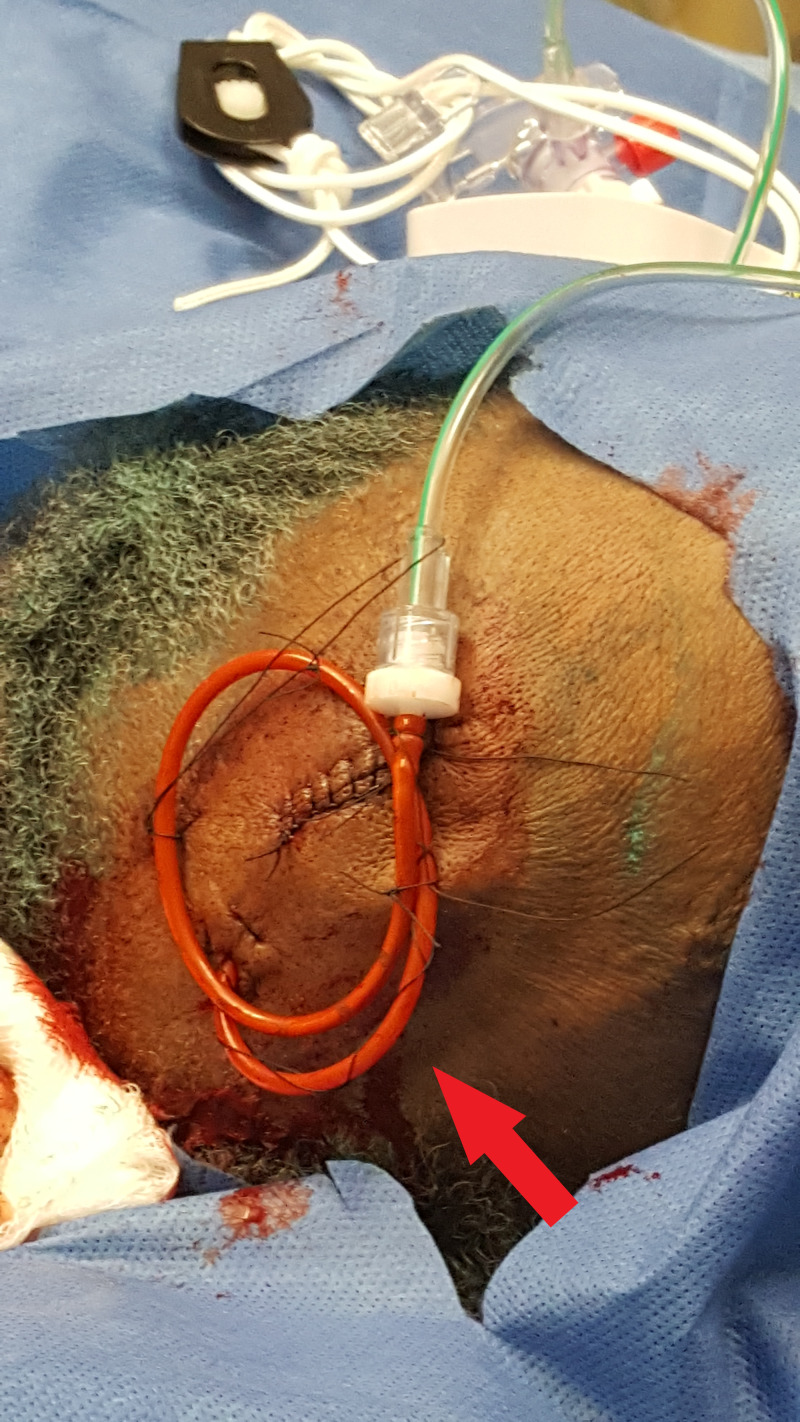
Demonstration of catheter securement to the scalp

**Figure 3:**
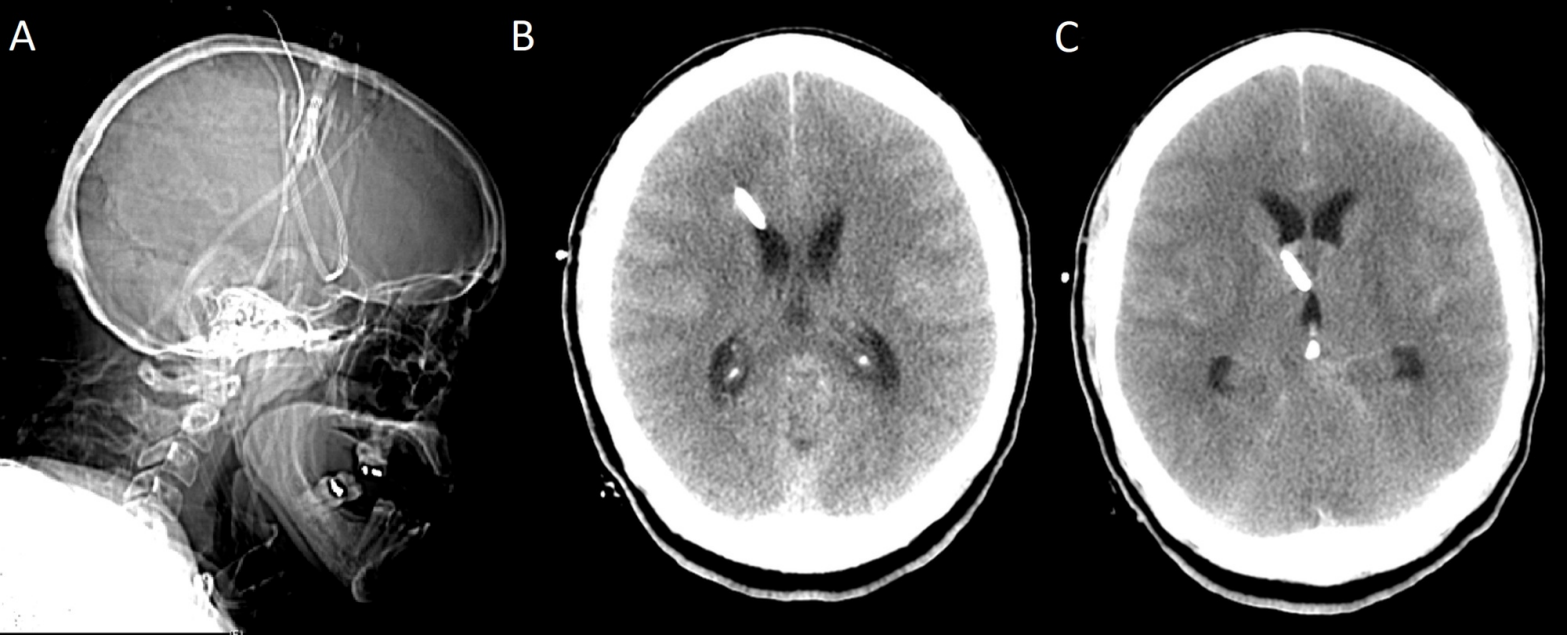
Sagittal scout image (A), Axial initial CT head scans following external ventricular drains (EVD) insertion (B and C) showing catheter tip terminating at Foramen of Monro

She was emergently transferred to our institution for neurosurgical care. On arrival, she was drowsy but arousable to stimulation. Her extraocular movements were intact at this time. CT angiography (CTA) revealed a left internal carotid artery (ICA) aneurysm. An EVD was placed bedside using the free-hand method and secured via a purse-string suture at the drain site then applied to the catheter in a “Roman sandal” fashion (Figure 2) without any issues within one hour of arrival and confirmed to be correctly positioned in the frontal horn of the right lateral ventricle (Figure 3).

She underwent coil-embolization of the aneurysm. During the procedure the EVD was open to drainage at 20 cm H_2_O. She underwent successful coil-embolization of the aneurysm and was able to be extubated. Her neurological examination improved and post-operatively the EVD was lowered to 5 cm H_2_O.

**Figure 4:**
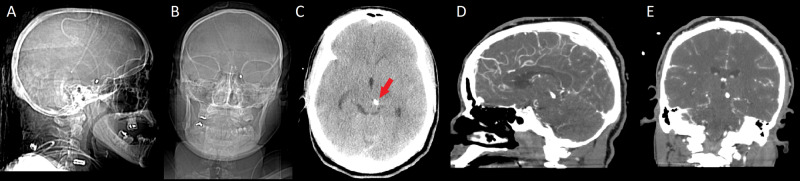
Sagittal scout (A), AP scout (B) Axial (C), Sagittal (D) and Coronal (E) CTA showing impingement of catheter tip on left tectum

Two days later, the patient complained of “shaky” vision, nausea and vomiting. She was found to have binocular vertical diplopia, bilateral superior gaze palsy, and induction of nystagmus and lid retraction on upgaze. CTA showed the EVD catheter migrated and traversed the third ventricle and terminated adjacent to the left dorsal midbrain (Figure 4). 

**Figure 5:**
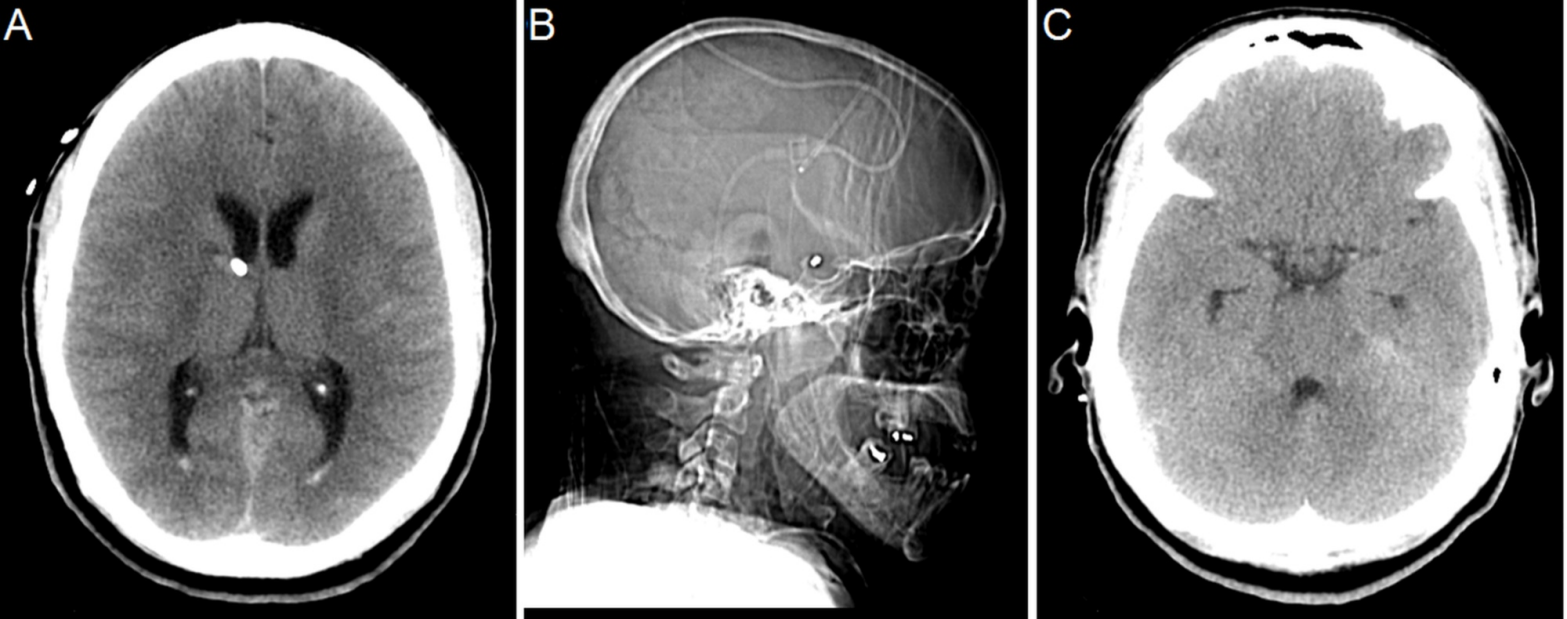
Axial CT head (A) and sagittal X-Ray (B) following adjustment of EVD showing the catheter tip in the Foramen of Monro. Axial CT head (C) showing no tectal blood following external ventricular drains (EVD) adjustment.

The EVD continued to function despite malposition. Inspection of the scalp at the securement site of the catheter showed the external portion was fixed and did not explain the inward migration. The EVD catheter was promptly readjusted and repeat CT head (CTH) confirmed correct placement with the distal catheter tip again repositioned in the frontal horn of the right lateral ventricle. The patient reported mild improvement in vision immediately after adjustment (Figure 5).

The patient’s Parinaud’s syndrome continued to improve daily both subjectively and per assessment by ophthalmology. The EVD was able to be weaned and removed. The patient was discharged home two days after the uneventful removal of the EVD.

## Discussion

There has only been one other report of an EVD causing Parinaud’s syndrome. In that case report, initial placement of the EVD was malpositioned at an outside institution. On arrival the patient already had an upward gaze palsy but the EVD was functional and her deficit was stable. Magnetic resonance image (MRI) of the patient’s brain showed the catheter tip in the tectum. The EVD was weaned and removed and the patient was discharged [4].

The malplacement of EVDs is well-discussed in the literature [5-11]. Placement by the free-hand method is estimated to have 70-79% positional accuracy and 86-88% functional accuracy [5,7,9,10]. Studies have found that the highest rates of suboptimal placement was in trauma patients and patients with midline shift, and the highest rate of optimal placement was in patients with SAH [8,9].

In the case of our patient, subsequent migration after initial placement caused impingement on the dorsal midbrain leading to Parinaud’s syndrome. This migration suggests that securing the catheter may have been the cause of the complication. The catheter was secured via a purse-string suture, which was then applied to the catheter in a “Roman sandal” fashion. Although this standard method of securement prevents migration of the catheter outwards, it is clearly not adequate to prevent inward migration. We suggest that the additional application of an adhesive at the exit site, such as an acrylate similar to Dermabond (Ethicon, Somerville, NJ, USA), may help prevent migration of the catheter in either direction. 

## Conclusions

This is a novel case report of an initially correctly placed EVD that migrated causing impingement on the dorsal midbrain. Using an adhesive at the exit site may have prevented the inward migration of the catheter. Vigilance in assessing for changes in neurological examination allowed for quick assessment of the location of the catheter tip, leading to prompt revision and subsequent recovery in this patient.
